# Poly(I:C) promotes TNFα/TNFR1-dependent oligodendrocyte death in mixed glial cultures

**DOI:** 10.1186/1742-2094-8-89

**Published:** 2011-08-03

**Authors:** Andrew J Steelman, Jianrong Li

**Affiliations:** 1 Department of Veterinary Integrative BioSciences, Texas A&M University, College Station, Texas, 77843, USA

**Keywords:** poly(I:C), oligodendrocyte death, TLR, TNFα, microglia activation, astrocytes

## Abstract

**Background:**

Activation of glial cells via toll-like receptors (TLRs) and other intracellular pathogen recognition receptors promotes the release of potentially toxic acute phase reactants such as TNFα and nitric oxide into the extracellular space. As such, prolonged glial activation, as is thought to occur during a persistent viral infection of the CNS, may contribute to both neurodegeneration and demyelination. However, the effects of virus-induced glial activation on oligodendrocytes are not fully understood.

**Method:**

To determine the effects of glial activation on oligodendrocyte viability we treated primary glial cultures isolated from neonatal rats or mice with the RNA viral mimic poly(I:C) and in some cases other TLR ligands. TLR3 expression was determined by western blot. Cytokine levels were measured by RT-PCR, ELISA, and intracellular cytokine staining. Oligodendrocyte precursor (preOL) viability was determined by Alamar blue assays and immunocytochemistry.

**Result:**

Stimulation of mixed glial cultures with poly(I:C) resulted in microglia activation, TNFα production and preOL toxicity. This toxic effect of poly(I:C) was indirect as it failed to affect preOL viability in pure cultures despite the fact that preOLs express TLR3. Poly(I:C)-induced loss of preOLs was abolished in TNFα or TNFR1 deficient mixed glial cultures, suggesting that TNFα/TNFR1 signaling is required for poly(I:C) toxicity. Furthermore, although both microglia and astrocytes express functional TLR3, only microglia produced TNFα in culture. Consistent with these findings, other TLR agonists similarly triggered TNFα production and preOL toxicity in mixed glial cultures.

**Conclusion:**

Activation of microglia by poly(I:C) promotes TNFα/TNFR1-dependent oligodendroglial cell death. These data indicate that during an ongoing viral infection of the CNS, microglial TNFα may be detrimental to oligodendrocytes.

## Background

The central nervous system (CNS) is considered immune privileged, but is not without a functional defense system [1]. Specifically, no organized lymphoid tissues or professional antigen presenting cells are thought to exist in the healthy CNS parenchyma. However, both resident microglia and astrocytes have been shown to possess immune surveillance properties and are thought to contribute to the chemoattraction and stimulation of antigen primed T-cells [2,3]. An innate ability to distinguish self from non-self is imperative for the limitation of pathogen spread, the stimulation of adaptive immune responses and the induction of tissue repair [4,5]. Toll-like receptors (TLR), nucleotide-oligomerization domain (NOD)-like receptors, and intracellular retinoic acid-inducible gene I (RIG-I)-like receptors all play an integral role in this process through the up-regulation of cytokines, chemokines, and costimulatory molecules following ligation of specific pathogen associated molecular patterns [6].

Several intracellular viral recognition receptors are constitutively expressed in the CNS and can be up-regulated upon stimulation [7]. Included in these receptors are TLR3 [8], RIG-I [9,10] and melanoma differentiation-associated protein-5 (MDA-5) which have been shown to be expressed in microglia and astrocytes [10,11] as well as neurons [8,12-14] and oligodendrocytes [15,16]. While the activation of TLR3 in astrocytes by RNA viral mimic polyinosinic:polycytidylic acid (poly(I:C)) causes the release of soluble molecules with neuroprotective properties [7], over-activation of TLRs in the absence of immunoregulation may be detrimental to the surrounding tissue [17-21].

Direct viral infections or aberrant inflammation consequent of previous infection are known to contribute to both animal and human demyelinating diseases [22]. For instance, experimental infection of animals with Theiler's murine encephalomyelitis, mouse hepatitis and Semliki Forest viruses cause demyelination of the CNS [22]. Also, multiple human demyelinating diseases can result from viral infection or vaccination, such as progressive multifocal leukoencephalopathy, human T-cell lymphotropic virus type I-associated myelopathy, subacute sclerosing panencephalitis and acute disseminated encephalomyelitis [22]. Viral infections have also been implicated in the onset of multiple sclerosis (MS), the most common demyelinating disease of the CNS [23]. Interestingly, TLR3 expression has been shown to be increased in both astrocytes and microglia in chronic MS lesions and is co-localized with its endogenous ligand stathmin, implicating a role for the activation of this particular TLR in MS pathogenesis [8].

Previous studies have demonstrated that lipopolysaccharide (LPS) and subsequent activation of TLR4 is capable of inducing oligodendrocyte death *in vivo *as well as *in vitro *[17] and that this cell death is dependent on both TNFα production [24] and TNFR1 signaling [25]. The stimulation of mixed glial cultures with LPS provides a relevant model with which to study certain CNS white matter pathology brought on by gram-negative bacteria, which are thought to contribute to periventricular leukomalacia, a major cause of cerebral palsy. However, the use of LPS *in vitro *may not accurately reflect inflammatory responses of glial cells during a viral infection; particularly since the pattern recognition receptors that recognize LPS and poly(I:C) are distinct and unequally distributed in parenchymal cells [15]. Direct viral infection of glial cultures provides a relevant tool to model CNS viral-induced pathogenesis and/or tropism. However, infection of CNS-derived cultures with live virus often makes it difficult to separate virus-induced cytopathic effect (*i.e*. cell lysis) from bystander effects of activated glia.

While poly(I:C) can activate both microglia [26] and astrocytes [27], the impact of this activation on oligodendrocyte viability has not yet been established. Herein, we demonstrate that poly(I:C) stimulation of mixed glial cultures results in a reduction of oligodendrocyte precursor (preOL) viability in a TNFα- and TNFR1-dependent mechanism. Interestingly, the effects of poly(I:C) stimulation were very similar to those induced by LPS stimulation, and suggest that without proper regulation of glial inflammatory responses, aberrant production of TNFα within the CNS can be detrimental to developing oligodendrocytes.

## Methods

### Mice and rats

Primary cells (mono-, co-, and mixed glial cultures as well as splenocytes) were isolated from Sprague Dawley rats (Harland; Houston TX). Mouse cells were obtained from wildtype B6.129SF2/J, C57BL/6J, and TNFα^-/- ^(background B6.129SF2/J) and TNFR1^-/- ^(background C57BL/6J) mice from The Jackson Laboratory (Bar Harbor, ME). Animals were housed under constant 12 h light/dark cycles in covered cages and fed with a standard rodent diet *ad libitum*. All experimental procedures were approved by the Institutional Animal Care and Use Committee and were performed in accordance with guidelines of the National Institutes of Health.

### Isolation of primary cells

Oligodendrocytes, microglia, astrocytes and mixed glial cultures were isolated from the forebrains of 1 to 2-d-old Sprague Dawley rats or mice using a differential detachment procedure [28]. Briefly, after brain dissection and removal of the meninges the forebrains were digested with HBSS containing 0.01% trypsin and 10 μg/ml DNase for 5 min at 37°C. The cells were washed twice with Dulbecco's Modified Eagle Media (DMEM) containing 10% heat-inactivated fetal bovine serum and 1% penicillin-streptomycin. Following filtration through a sterile 70 μm filter to remove excess debris, the cells were counted and plated onto poly-d-lysine coated 75 cm^2 ^flasks or directly into 24-well plates for experiments using mixed glia. For mixed glial culture experiments, the medium was changed every other day and cells were treated after 7-8 days *in vitro *(DIV) in serum-free Basal Defined Medium (BDM: DMEM containing 0.1% bovine serum albumin, 50 μg/ml human apo-transferrin, 50 μg/ml insulin, 30 nM sodium selenite, 10 nM D-biotin, and 10 nM hydrocortisone). For experiments involving mono and co-cultures, microglia were isolated by shaking the mixed glia-containing flasks for 1 h at 200 rpm. After removing microglia, the flasks were shaken overnight to separate preOLs from the astrocyte layer. Single cell suspensions from rat spleens [29] were used as a positive control for TLR3 western blot analysis. Briefly, the external capsule of the spleen was broken and cells passed through sterile 70 μm nylon mesh twice in DMEM containing 10% FBS. The cells were pelleted by centrifugation at 1500 × *g *for 5 min and the red blood cells were lysed with ammonium chloride (0.01 M) in PBS for 3 min at 24°C. The splenocytes were then washed in PBS.

### Immunocytochemistry

Cells were fixed with 4% paraformaldehyde (PFA) in PBS (pH 7.4) for 10 min at room temperature, blocked with 5% goat serum in PBS for 1 h at room temperature and incubated with the O4 antibody (1:500) overnight at 4°C. After washing with PBS, they were incubated with fluorescence conjugated goat anti-mouse IgM (Invitrogen, Carlsbad, CA) for 1 h. Following washing they were post-fixed with 4% PFA for 10 min, and blocked and permeabilized with 5% goat serum in TBS containing 0.1% Triton-X 100 (TBST) for 1 h, and then incubated with either anti-Iba1 (1:500, rabbit IgG; Wako, Richmond, VA) or anti-GFAP (1:100, rabbit IgG; Invitrogen, Carlsbad, CA) overnight at 4°C. The cells were incubated with fluorescence conjugated goat anti-rabbit IgG (Invitrogen, Carlsbad, CA) for 1 h at room temperature, and counter stained with Hoechst (Invitrogen, Carlsbad, CA) as described previously [24].

### TNFα measurement and intracellular cytokine staining

Rat and mouse TNFα levels were measured from supernatants by ELISA kits according to the manufacturer's instructions (eBioscience; San Diego, CA.). Intracellular cytokine staining of TNFα was performed as described previously [25]. Briefly, mixed glial cultures were washed twice with media then treated with media, PBS, poly(I:C) (50 μg/ml; Sigma, St. Louis, MO) or LPS (1.0 μg/ml; Sigma, St. Louis, MO) for 8 or 24 h at 37°C. During the last 5 h of stimulation, protein secretion was inhibited using Brefeldin-A (eBioscience; San Diego, CA.) diluted 1:1000 in BDM alone or in BDM containing poly(I:C) or LPS. The cells were then fixed and immunostained as described above.

### RT-PCR

Transcriptional expression of Iba-1, GFAP, TLR3, TNFα, IL-1β, and IL-6 in microglia and astrocyte monocultures upon stimulation with poly(I:C) was examined as described previously [25]. Briefly, cells were plated onto poly-D-lysine coated 6-well plates at a density of 1.0 × 10^6 ^cells per well in DMEM containing 10% fetal bovine serum. After 24 h, cells were washed twice with BDM medium, and then treated with poly(I:C) (50 μg/ml) or vehicle control (PBS) for 0-24 h. RNeasy kits were used to extract RNA according to the manufacturers' instructions (Qiagen, Valencia, CA). Residual DNA was digested by incubating RNA samples with DNase I for 15 min at room temperature followed by DNase inactivation at 65°C for 10 min according to the manufacturer's instructions (Invitrogen, Carlsbad, CA). The samples were reverse transcribed to cDNA using the reverse transcription system kit (Promega, Madison, WI) as described previously [25]. The purity of the mono-cultures was determined using specific primers for GFAP (astrocyte marker) and Iba-1 (microglia marker). Primers specific for β-actin were used as a loading control. All products were amplified by PCR using 100 ng of cDNA and the following primers: TLR3, forward-TGCGATTGGCAAGTTATTCG, reverse-GCGGAGGCTGTTGTAGGAAA; TNF, forward-GCCCACGTCGTAGCAAAC, reverse-GCAGCCTTGTCCCTTGAA; IL-1β, forward-TGACCCATGTGAGCTGAAAG, reverse-AGGGATTTTGTCGTTGCTTG; IL-6, forward-CAGGAACGAAAGTCAACTCCA, reverse-ATCAGTCCCAAGAAGGCAACT; CCL2, forward-CCAGCCCAGAAACCAGCCAACTC, reverse-GCATCTGGCTGAGACAGCACGT; CCL5, forward-AGCAGCAAGTGCTCCAACCTTG, reverse-GCACACCTCCCAGGCCATAGGA; GFAP, forward-CAGCTT CGAGCCAAGGAG, reverse-TGTCCCTCTCCACCTCCA; Iba1, forward-CTTTTGGACTGCTGAAAGCC, reverse-GTT TCTCCAGCATTCGCTTC; and β-actin, forward-AGACTTCGAGCAGGAGATGG, reverse-CCATCATGAAGTGTGACGTTG. Products were electrophorezed on 2% agarose gels and visualized under UV light using a Bio-Rad Chemidoc XRS gel documentation system and Quantity-one software. Quantitative PCR was used to determine fold increase in CCL2 and CCL5 relative to β-actin using the formula 2^-ΔΔCt ^as described previously [30].

### Cell Viability Assays

Cell viability following TLR agonist treatment was assessed using both immunocytochemical and biochemical techniques. Lactate dehydrogenase (LDH) activity was assessed from culture supernatants according to the manufacturer's instructions (Roche Applied Science; Indianapolis, IN). The effects of treatment on preOL viability in mixed glial cultures were determined 48 h after treatment with Pam3CSK4 (1.0 μg/ml; InvivoGen, San Diego, CA), poly(I:C) (50 μg/ml and 5 μg/ml), LPS (1.0 and 0.1 μg/ml) or R848 (5.0 μg/ml; InvivoGen, San Diego, CA) by counting the number of normal appearing nucleated O4^+ ^cells per 200× magnification from 5 random fields per culture well. O4^- ^cells most likely representing microglia and astrocytes were excluded from the analysis. Pictures were taken using an Olympus DP70 digital camera mounted on an Olympus IX71 microscope. All treatments were conducted in at least triplicate, corresponding to between 750-1000 cells counted per treatment condition per experiment. Experiments were replicated at least three times. For each experiment the average number of O4^+ ^cells in the control group was used to obtain percent reduction, and the percent reduction averaged across experiments. The effects of various treatments in pure preOL cultures were assessed by Alamar blue assay as described previously [24].

### Western blot

Cell lysates from microglia, astrocyte and oligodendrocyte monocultures as well as splenocytes were used to determine the presence of TLR3 protein. Briefly, cells were lysed on ice in standard lysis buffer (10 mM Na_2_P_2_O_7_, 10 mM NaF, 100 mM Na_3_VO_4 _100 mM PMSF proteinase inhibitor cocktail; Roche) and then sonicated. Cell debris was pelleted by centrifugation at 16000 × *g *for 15 min at 4°C. Total proteins (20 μg) were separated by SDS-polyacrylamide gel electrophoresis followed by transferring to PVDF membranes. After blocking with TBS-T (50 mM Tris-HCl, pH 7.4, 150 mM NaCl, 0.1% Tween-20) containing 5% non-fat milk for 1 h at RT the membranes were incubated with anti-TLR3 (1: 200, Rabbit IgG, Santa Cruz Biotech, Santa Cruz, CA.) overnight at 4°C or β-actin (1:10,000, Mouse IgG, Sigma, St. Louis, MO) diluted in TBS-T containing 5% non-fat milk. The membranes were washed 3-5 times with TBS-T, and then incubated with horseradish peroxidase conjugated secondary antibody for 1 h. Following additional washes bands were detected by chemiluminescence using the SuperSignal detection kit (Thermo Scientific, Rockford, IL).

### Statistics

All data are expressed as means ± SEM. Data were analyzed using two-tailed student's t-tests for comparisons between two groups or multiple level analysis of variance (ANOVA) followed by Bonferroni's post-hoc test where appropriate to distinguish differences between multiple groups. All statistics were calculated using GraphPad Prism 4 (GraphPad Software, San Diego, CA). Differences were considered significant when *p *< 0.05.

## Results

### Poly(I:C) activates microglia and induces pre-oligodendrocyte cell death in mixed glial cultures

**Figure 1:**
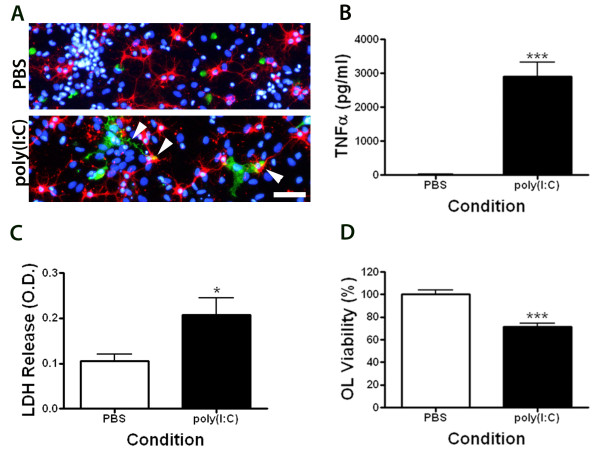
**Stimulation of mixed glial cultures with poly(I:C) causes microglia activation and preOL cell death**. Rat mixed glial cultures were stimulated with vehicle (sterile PBS; 5 μl/ml) or poly(I:C) (50 μg/ml) in BDM for 48 h. ***A: ***Immunocytochemical staining showing changes in microglia morphology (Iba-1; green) with process extensions closely associated with O4^+ ^preOLs (red) in poly(I:C) treated cultures (arrows). Scale bar, 50 μm. ***B, C: ***Poly(I:C) stimulated cultures had elevated TNFα **(B) **and LDH **(C) **in culture supernatants as determined by ELISA and LDH assays respectively. ***D***. O4^+ ^cell viability was decreased following in poly(I:C) treatment. Results in ***A ***are representative of 3 independent experiments. Results from ***B-D ***are combined means ± SEM of 4-5 independent experiments.

**Figure 2:**
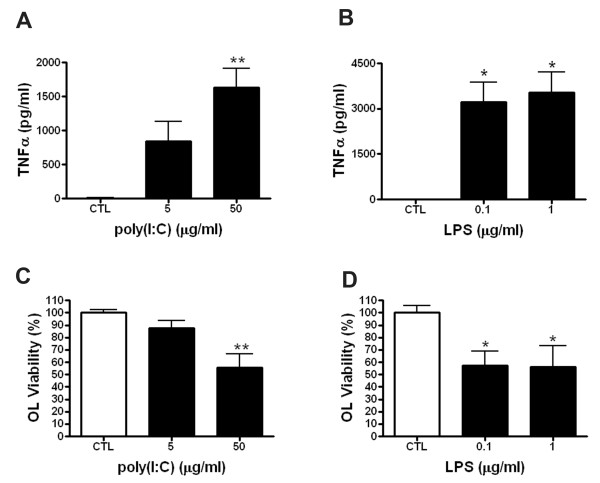
**Poly(I:C) mediated reduction in preOL viability is dose-dependent**. Rat mixed glial cultures were stimulated with vehicle (sterile PBS; 5 μl/ml), poly(I:C) (5 and 50 μg/ml), or LPS (positive control; 0.1 and 1.0 μg/ml) for 48 h. ***A, B*: **Poly(I:C) dose-dependently increased TNFα levels as measured by ELISA (**A**) and reduced preOL viability at 50 μg/ml but not 5 μg/ml (**B**). ***C, D: ***Stimulation of mixed glia with 0.1 and 1.0 μg/ml of LPS induced similar levels of TNFα production (**C**) and loss of preOL viability (**D**). Results are combined means ± SEM from 3 independent experiments. *, *p *< 0.05, **, *p *< 0.01.

Treatment of mixed glial cultures with poly(I:C) caused robust microglial activation as indicated by marked changes in morphology (Figure 1A), TNFα secretion (Figure 1B) and increased cell death as determined by LDH release 48 h after the treatment (Figure 1C). Processes of poly(I:C)-activated microglia were frequently found to be in close proximity to preOLs, suggesting an increase in their surveillance properties (Figure 1A, arrow). Immunocytochemical analysis revealed an approximate 28% loss of preOLs in the poly(I:C) stimulated cultures when compared to control cultures (Figure 1D). Furthermore, poly(I:C) reduced preOL viability in a dose-dependent manner that appeared to correlate with TNF production (Figure 2A-D). These data indicate that poly(I:C) stimulation in mixed glial cultures is toxic to preOLs.

### Poly(I:C) is not directly toxic to pre-oligodendrocytes

**Figure 3:**
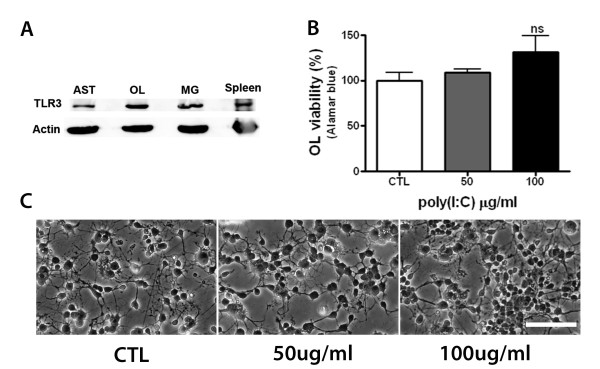
**Poly(I:C) stimulation is not directly toxic to preOLs**. ***A: ***Western blot analysis of TLR3 expression in astrocyte (AST), oligodendrocytes (OL), and microglia (MG). Splenocytes were used as a positive control. ***B: ***Poly(I:C) did not cause cell death in pure preOL cultures. PreOLs were treated with increasing concentrations of poly(I:C) for 48 h and cell viability measured by Alamar blue. Results are combined means ± SEM of 4 independent experiments; ns, not significant. ***C: ***Representative phase-contrast images of preOLs treated as above. Scale bar, 100 μm.

As oligodendrocytes, astrocytes and microglia reportedly express TLR3 [7,15], a prominent receptor for poly(I:C), it was possible that preOLs in mixed glial cultures were dying in a cell autonomous fashion in response to poly(I:C). Consistent with previous reports, western blot analysis revealed that all glial cells expressed TLR3 in culture (Figure 3A). To examine the direct effects of poly(I:C) on preOLs, purified preOL cultures were stimulated with increasing concentrations of poly(I:C) and cell viability was determined. In contrast to poly(I:C)-induced preOL death in mixed glial cultures, we found that poly(I:C) was not toxic to pure preOLs (Figure 3B-C). Instead, poly(I:C) treatment appeared to result in increased viability, but this effect was not statistically significant.

### TNFα and its cognate receptor R1 are responsible for pre-oligodendrocyte cell death following poly(I:C) stimulation in mixed glia cultures

**Figure 4:**
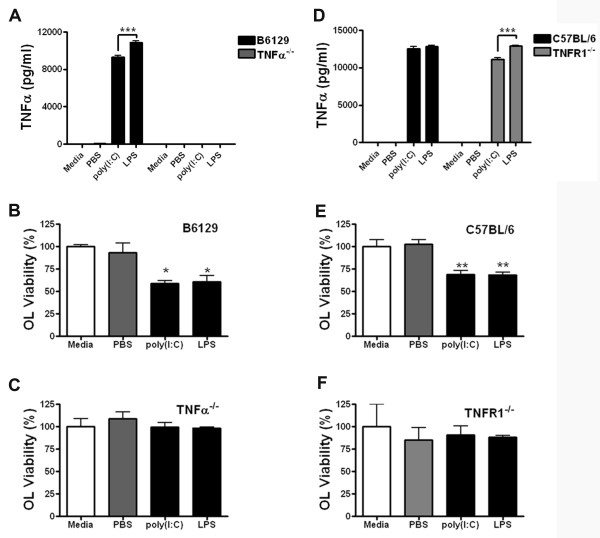
**Both TNFα production and TNFR1 are required for poly(I:C)-induced preOL death in mixed glial cultures**. Mixed glial cultures derived from WT, TNF^-/- ^or TNFR1^-/- ^were stimulated with vehicle (sterile PBS; 5 μl/ml), poly(I:C) (50 μg/ml), or LPS (1.0 μg/ml) for 48 h. ***A, D: ***TNFα production as determined by ELISA. Results are means ± SEM of 3 independent experiments. ***B-C; E-F: ***PreOL viability as determined by O4^+ ^cell counting. Both LPS and poly(I:C) caused preOL toxicity in cultures derived from WT B6129 **(B) **and WT C57BL/6 **(E) **mice, but had no effect in cultures derived from TNF^-/- ^**(C) **or TNFR1^-/- ^**(F) **mice. Results are means ± SEM of 3 independent experiments for each mouse strain. *, *p *< 0.05, **, *p *< 0.01, ***, *p *< 0.001.

To test whether the detrimental effects of poly(I:C) were attributable to TNFα production, we prepared mixed glial cultures from TNFα^-/-^, TNFR1^-/- ^and their corresponding wildtype (WT) mice. As we previously found that both TNFα production and TNFR1 signaling are required for LPS-induced preOL cytotoxicity in mixed glial cultures, we used LPS as a positive control [24,25]. Similar to LPS, poly(I:C) induced significant TNFα production in B6129 (WT), C57BL/6 (WT) and TNFR1^-/- ^, but not in TNFα^-/- ^mixed glial cultures (Figure 4A&4D). TNFα production from poly(I:C) stimulated cultures was generally lower than those stimulated with LPS, an effect that was significant in B6129 and TNFR1^-/- ^cultures (Figure 4A&4D).

To determine if preOL viability decreased upon poly(I:C) stimulation in various cultures we counted the number of preOLs under each condition. Consistent with the above data, stimulation of WT cultures with poly(I:C) or LPS resulted in significant loss of O4^+ ^cells compared to vehicle control treated cultures (Figure 4B&4E). In contrast, this cytotoxic effect was abolished in TNFα deficient mixed glial cultures (Figure 4C). Deletion of the TNFα receptor *tnfr1 *also abrogated the poly(I:C)-triggered TNF-mediated toxicity to preOLs (Figure 4F) despite a comparable level of TNFα production in WT and TNFR1^-/- ^cultures (Figure 4D). These results demonstrate that TNFα is required for oligodendroglial cell death in mixed cultures challenged with poly(I:C) or LPS and that the signal needed to induce cell death is dependent on TNFR1.

### Microglia are the sole source of TNFα secretion after poly(I:C) stimulation in culture

**Figure 5:**
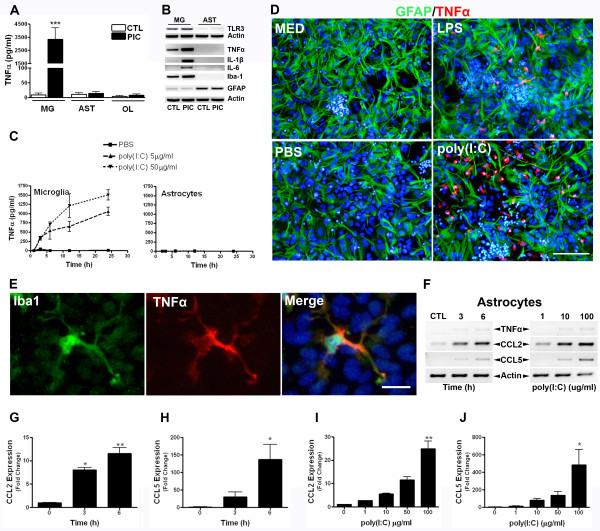
**Microglia produce TNFα following poly(I:C) stimulation**. ***A: ***Microglia (MG), astrocytes (AST) or oligodendrocytes (OL) were treated with or without poly(I:C) (PIC; 50 μg/ml) for 24 h and TNFα levels were determined by ELISA. Results are means ± SEM of 3 independent experiments. ***B: ***Expression of TLR3 (top), TNFα, IL-1β, IL-6, Iba-1 and GFAP (bottom) was determined by RT-PCR from monocultures treated with PBS or poly(I:C) for 24 h. Results are from 2 independent experiments and are representative of 3 independent experiments for each product. ***C: ***Kinetic analysis of TNFα production following stimulation with varying doses of poly(I:C) in microglia (left) and astrocyte (right) monocultures. Results are combined means ± SEM of triplicate wells and are representative of 3 independent experiments. ***D, E: ***Intracellular cytokine staining of mixed glial cultures treated with vehicle (sterile PBS; 5 μl/ml), poly(I:C) or LPS for 8 h. TNFα immunoreactivity did not localize to GFAP^+ ^astrocytes **(D)**, but instead to Iba-1^+ ^microglia **(E)**. Results are representative of 3 independent experiments. ***F: ***Expression of TNFα, CCL2 and CCL5 was determined by RT-PCR from astrocytes treated with vehicle (sterile PBS; 5 μl/ml) or poly(I:C) (50 μg/ml) for 3-6 h (left) or increasing concentrations (right) of poly(I:C) for 6 h. Results are representative of 2 independent experiments. ***G-J: ***Both CCL2 **(G, I) **and CCL5 **(H, J) **expression were dose- and time-dependently increased upon poly(I:C) stimulation. Results are means ± SEM from duplicate (CCL2) or triplicate (CCL5) samples and are representative of 3 independent experiments. *, *p *< 0.05, **, *p *< 0.01, ***, *p *< 0.001.

Both microglia and astrocytes are capable of cytokine and chemokine secretion following recognition of various pathogen associated molecular patterns including poly(I:C). To determine the cellular source of TNFα secretion in our cultures, purified microglia, astrocytes or oligodendrocytes were treated with PBS or poly(I:C) for 24 h and TNFα levels were determined by ELISA. We found that microglia, but neither astrocytes nor oligodendrocytes, produced TNFα following poly(I:C) stimulation (Figure 5A). To confirm this finding, we then conducted RT-PCR analysis on RNA isolated from microglia and astrocyte monocultures stimulated with or without poly(I:C) for 24 h. Both microglia and astrocytes expressed TLR3. TNFα and IL-1β transcripts were induced by poly(I:C) in microglia, but undetectable in astrocytes. Moreover, transcription of IL-6 was evident in stimulated microglia and in poly(I:C) stimulated astrocytes (Figure 5B). The expression of the macrophage/microglia marker Iba-1 and astrocyte marker GFAP from the same samples indicated that there was virtually no microglia contamination of astrocyte cultures, but some astrocyte contamination in microglia cultures (Figure 5B). Additionally, microglia but not astrocytes responded to poly(I:C) by producing TNFα in a dose- and time-dependent fashion (Figure 5C). These results strongly indicate that microglia are the producers of TNFα in mixed glial cultures.

To further determine whether microglia were indeed the TNFα producers and to test whether microglia-derived factors were capable of inducing astroglial TNFα, we performed intracellular cytokine staining for TNFα in mixed cultures treated with poly(I:C). We found that TNFα^+ ^cells with microglia morphology were abundant upon stimulation with poly(I:C) or LPS, but were absent in vehicle control treated mixed glial cultures (Figure 5D). Immunostaining of poly(I:C)-stimulated cultures with an isotype control antibody did not reveal any non-specific signal (not shown). Importantly, dual staining for TNFα and either GFAP or Iba-1 demonstrated that astrocytes did not produce TNFα following stimulation with LPS or poly(I:C) as TNFα^+ ^cells never localized to GFAP^+ ^cells (Figure 5D). Instead, TNFα immunoreactivity was confined to Iba-1^+ ^cells, indicating microglia as being the sole source of TNFα under these conditions (Figure 5E).

Our observation that astrocytes express TLR3, yet did not produce TNFα following poly(I:C) stimulation raised the possibility that poly(I:C) was incapable of activating astrocytes under our experimental paradigms. To test whether exogenous poly(I:C) was sufficient to elicit a response from astrocytes, we next determined the expression of chemokines CCL2 and CCL5, which have been previously reported to be substantially induced in astrocytes following extracellular poly(I:C) stimulation [11,27]. Again, while stimulation of astrocytes with poly(I:C) resulted in negligible TNFα transcription we observed marked up-regulation of both CCL2 and CCL5 in a time- and dose-dependent fashion (Figure 5F), which was further confirmed by qRT-PCR analyses (Figure 5G-J). These results indicate that astrocytes indeed respond to poly(I:C) and produce chemokines while at the same time fail to transcribe TNFα.

### Stimulation of mixed glial cultures with other TLR ligands causes toxicity to preOLs

**Figure 6:**
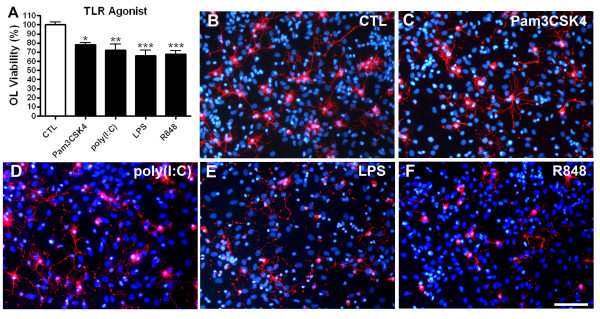
**Stimulation of mixed glia with TLR2 and TLR7 agonists increases TNFα and promotes preOL cytotoxicity**. Rat mixed glial cultures were treated with vehicle (sterile PBS; 5 μl/ml), TLR1/2 agonist Pam3CSK4 (1.0 μg/ml), TLR3 agonist poly(I:C) (50 μg/ml), TLR4 agonist LPS (1.0 μg/ml) or TLR7/8 agonist R848 (5.0 μg/ml) for 48 h. PreOL viability was determined by counting O4^+ ^cells. Scale bar, 50 μm. Results are combined means ± SEM from 4 independent experiments. *, *p *< 0.05, **, *p *< 0.01, ***, *p *< 0.001.

Our above data demonstrate that stimulation of mixed glial cultures with poly(I:C), a mimic of double-stranded RNA viral infection, is toxic to preOLs. Similar to what we have shown previously after LPS challenge [24,25], the toxicity triggered by poly(I:C) is also dependent on the TNFα/TNFR1 pathway. Because ligation of other TLRs on microglia can also induce TNFα production [31], we questioned whether stimulation of mixed glia with additional TLR ligands could also result in reduced preOL viability. Indeed, stimulation of mixed cultures with TLR1/2 agonist Pam3CSK4 or TLR7/8 agonist R848 triggered TNFα production (Table 1) with LPS stimulation causing significantly more TNFα production than any other TLR agonists tested. All tested TLR agonists resulted in a significant reduction in O4^+ ^cell viability that was most pronounced following treatment with LPS and R848 (Figure 6). Together, these data support a role for aberrant TNFα production in mediating preOL death.

**Table 1:** TNFα production in mixed glial cultures stimulated with TLR agonists

Stimulus	TLR ligand	TNFα (pg/ml)	Significance
Control	N/A	36.14 ± 3.112	
Pam3CSK4	TLR1/2	1865 ± 353.5	*p *< 0.01
poly(I:C)	TLR3	1420 ± 73.39	*p *< 0.05
LPS	TLR4	4645 ± 214.4*	*p *< 0.001
R848	TLR7/8	1972 ± 361.1	*p *< 0.001

Mixed glia were stimulated for 48 h with various TLR ligands and TNFα measured from culture supernatants by ELISA. Results are combined means ± SEM from 4 independent experiments. *LPS stimulation resulted in significantly higher TNFα production compared to all other TLR ligands (*p *'s < 0.001).

## Discussion

In the current study we demonstrate that stimulation of mixed glial cultures with the viral mimic poly(I:C) causes preOL death in a non-cell autonomous fashion and through a TNFα/TNFR1-dependent mechanism. TNFα was derived exclusively from activated microglia as astrocytes neither secreted TNFα nor expressed intracellular TNFα following poly(I:C) stimulation. This was surprising as astrocytes clearly express TLR3 [7,15,32-34], RIG-I [10,35] and MDA-5 [11] and have been shown to produce TNFα *in vivo *[36-39]. As these receptors are predominantly intracellular, a plausible explanation for these results could be the exogenous manner of the treatment. However, the fact that astrocytes up-regulated chemokines CCL2 and CCL5, but failed to induce TNFα indicates that exogenous poly(I:C) is capable of activating astrocytes thereby excluding the above possibility. In line with this finding, several recent studies have demonstrated that astrocytes stimulated with exogenous poly(I:C) dramatically increase cytokines IL-6, IFN-β, IL-8 and chemokine CCL2 and CCL5, but produce only negligible amounts of TNFα [11,27]. Moreover, flow cytometric analysis by Zhou et al. convincingly demonstrates that while astrocytes express CCL2 following Pam3CSK4, poly(I:C) or LPS stimulation or LCMV infection, they do not express TNFα [40]. Together, these results strongly indicate either that, microglia are far superior at TNFα production when compared to astrocytes *in vitro *or that TNFα production in astrocyte cultures is attributable to residual microglia.

To date several studies have demonstrated reduced preOL viability following LPS stimulation of microglia/preOL co-cultures [28] or mixed glial cultures containing astrocytes [17,24,25,41]. Mechanistically, it has been shown that NO-derived peroxynitrite is required for mediating toxicity to preOLs in microglia/preOL co-cultures [28] and may indeed participate in the initial reduction of preOL viability (after 24 h) in mixed cultures [41]. However, 48 h of LPS stimulation in mixed glial cultures results in a loss of preOL viability that is attributable to TNFα [24,25,41]. As with other studies, the current set of experiments implicates aberrant TNFα production in the demise of oligodendrocytes [17,24,41,42], but the exact role of TNFα in the pathogenesis of demyelination *in vivo *are not yet fully understood. For instance, stimulation of mixed glial cultures with LPS results in TNFα production, which is toxic to oligodendrocytes *in vitro *[17,24,43]. TNFα has also been shown to be directly toxic to oligodendrocytes *in vitro *[44]. Moreover, in transgenic mice in which astrocytes express transmembrane TNFα demonstrate oligodendrocyte death and develop demyelination [42]. In agreement with this observation, we have shown that both astrocytes and oligodendroglial TNFR1 are required for TNFα-mediated oligodendrocyte toxicity *in vitro *[25]. Furthermore, direct cell-cell contact between astrocytes and oligodendrocytes mediates the TNFα toxicity. Therefore, accumulating data suggest that microglia as well as astrocytes are required for TLR agonist induced preOL toxicity.

It is important to note that the mixed glial cultures used in this study did not contain neurons. As functional neurons constitutively express several immunoregulatory molecules such as CD200 [45] and CX3CL1 [46] that are capable of modulating microglia activation and cytokine production, it is possible that stimulation of mixed neuronal cultures with poly(I:C) might mitigate the toxic effect on preOL. Thus, future experiments are needed to examine the effect of neurons on microglia inflammatory responses and preOL viability. However, several lines of evidence indicate that TLR ligation can contribute to tissue destruction within the CNS. For instance, stereotaxic injection of zymosan, a TLR2 ligand, into either the corpus callosum [20] or spinal cord [21] causes microglia activation and demyelination. Likewise, stereotaxic injection of LPS into the spinal cord induces demyelination [19]. Moreover, poly(I:C) injection into the substantia nigra pars compacta was shown to trigger microglia activation and neurodegeneration at high doses or susceptibility to neurodegeneration at subtoxic levels [18]. While these studies suggest that acute microglia activation subsequent to TLR ligation is detrimental to the surrounding tissues, certainly not all microglial responses result in neurodegeneration. Some can in fact promote tissue repair [4]. For instance, intravitreal injection of zymosan is associated with an increase in TGFβ1 and IL-1β production and induces myelination of retina [47]; and LPS treatment concurrent to detergent ethidium bromide-induced demyelination promotes repair, possibly through the recruitment of oligodendrocyte progenitors to the lesion [48].

Likewise, not all studies have assigned a detrimental role to TNFα in the CNS. For example, while injection of recombinant TNFα into the optic nerve resulted in demyelination [49], repeated intracerebral injection of TNFα resulted in hemorrhage, reactive gliosis and infiltration of mononuclear and polymorphonuclear cells but appeared to have no cytolytic effects [50]. Moreover, in the cuprizone model of toxic demyelination, which is characterized by extensive reactive gliosis, TNFα^-/- ^mice exhibit delayed demyelination but also impaired remyelination [36]. In addition, it has recently been shown that the resolution of pathology following virus-induced encephalitis requires TNFα for repair in the striatum and the hippocampus through TNFR1 and TNFR2 respectively [51]. On the other hand, Theiler's murine encephalomyelitis virus infection of TNFR1^-/- ^were found to exhibit up to 80% fewer seizures during virus-induced encephalitis when compared to control mice [52].

Ambiguous effects of TNFα have also been demonstrated in multiple sclerosis. Elevated TNFα levels in the cerebral spinal fluid of MS patients precede exacerbation and correlate with disease progression [39]. TNFα immunoreactivity has been localized to astrocytes on the lesion edge but not in the lesion center of MS patients [37]. Furthermore, increased expression of TNFR1 and TNFR2 in oligodendrocytes at the edge of chronic active lesions was reported [53,54]. While these findings suggest an involvement for TNFα in the pathogenesis of MS, treatment with monoclonal neutralizing anti-TNFα antibodies as well as neutralizing soluble receptors has, paradoxically, resulted in MS disease exacerbation [55,56]. Even more alarming, anti-TNFα therapy in rheumatoid arthritis patients has been associated with the onset of monophasic demyelination including optic neuritis that subsided after treatment withdrawal [57-60] and with the onset of MS [57,61]. As the capacity for TNFα therapy to neutralize CNS TNFα was not directly examined, these data may imply that peripheral TNFα is somehow required for maintaining demyelinating disease quiescence. Although the underlying mechanism remains unknown the recent finding that increased susceptibility to MS is associated with a single nucleotide polymorphism within the sixth intron of the *tnfr1 *gene calls upon further investigation as to the role the TNFα/TNFR1 pathway in this disease [62,63].

## Conclusion

Toll-like receptor expression is not equally represented across different populations of resident CNS cells. Since TLR3, a recognition receptor for double-stranded RNA viruses, is present in all glia, the current study tested the hypothesis that stimulation of mixed glia with the TLR3 ligand poly(I:C) would exhibit different outcomes than those following LPS stimulation. Utilizing poly(I:C) to model the bystander effects of glial activation on preOL viability following RNA viral infection, we found that over-activation of glia, specifically microglia, reduced preOL viability in mixed glial cultures. Mechanistically, this toxicity was dependent on TNFα/TNFR1 signaling. Extending on these findings we observed that activation of TLR1/2 and TLR7/8 had similar effects on preOLs. Thus, our data indicate that uncontrolled production of TNFα in the CNS upon viral infection may consequentially contribute to oligodendrocyte death and demyelination.

## Abbreviations

Poly(I:C): polyinosinic:polycytidylic acid; LPS: lipopolysaccharide; MS: multiple sclerosis; OLs: oligodendrocytes; preOLs: oligodendrocyte precursors; TLR: Toll-like receptor; TNFα: tumor necrosis factor alpha; TNFR1: TNFα receptor 1.

## Competing interests

The authors declare that they have no competing interests.

## Authors' contributions

JL and AJS designed the study. AJS carried out all the experiments. AJS and JL analyzed the data and wrote the manuscript. JL supervised the study and secured the funding. All authors read and approved the final manuscript.
